# A psychotic experience during adolescence: reasoning about differential diagnosis. Case report

**DOI:** 10.1590/1516-3180.2016.0307240317

**Published:** 2017-08-21

**Authors:** Vítor Ferreira Leite, Carla Andrade Araújo

**Affiliations:** I Medical Doctor, Master and Senior Resident of Child and Adolescent Psychiatry, Pediatric Psychiatry Service, Coimbra Pediatric Hospital, Centro Hospitalar e Universitário de Coimbra, Coimbra, Portugal.

**Keywords:** Psychotic disorders, Conversion disorder, Schizophrenia, Hallucination, Child psychiatry, Adolescent psychiatry

## Abstract

**CONTEXT:**

The aim of the present clinical review was to illustrate the diagnostic difficulty associated with psychotic experiences during adolescence, in the light of the multiplicity of circumstances interplaying during this period. It was also intended to illustrate the observation that not all hallucinations occur in the context of a declared psychotic disorder.

**CASE REPORT:**

The patient was a 16-year-old adolescent girl who came to the Emergency Department of Coimbra Pediatric Hospital. On admission, she displayed mood and sensory perception disorders, with a bizarre gait abnormality. A diagnosis of conversion disorder was finally suggested, in accordance with the International Classification of Diseases, 10^th^ edition.

**CONCLUSIONS:**

Conversive hallucinations are rare in the psychiatric literature. This diagnostic hypothesis only gained consistency over a long period of follow-up within a child and adolescent psychiatry outpatient service, which was fundamental for appropriate diagnostic clarification. The authors discuss psychotic experiences that can arise from a neurotic setting and share the reasoning that was constructed in relation to the differential diagnosis. The psychogenesis and phenomenology of this young patient’s conversive hallucinations and the therapeutic strategies adopted over the course of the follow-up are also discussed.

## INTRODUCTION

Since there is no consensus regarding the definition of psychosis, one can try to define it, in general terms, as a disturbance of thought and sensory perception that negatively affects behavior and overall functioning.^1^ Psychotic symptoms arise from different disorders and etiologies, as nonspecific phenomena.^2^^,^^3^^,^^4^ The most pervasive of these are perhaps delusions, hallucinations, disorganization of thought, speech and behavior, and negative symptoms (blunted affect, alogia and avolition).^1^^,^^2^^,^^3^ Minor psychotic symptoms are reported relatively often in the general population without, however, meeting the criteria for a clinical diagnosis of psychosis.^5^ The most commonly described prodromal manifestations of a first psychotic episode are reduced attention and concentration, anergy, decreased will and motivation, depressed mood, sleep disturbances, anxiety, social withdrawal, distrust, irritability and deterioration of the ability to function.^6^^,^^7^^,^^8^

Studies conducted among adolescents have found that the prevalence of “psychotic experiences” in this age group is between 17 and 18%.^1^ These are frequently associated with states of emotional disturbances (mood or anxiety disorders) and it has been stated that in 90% of these cases, no declared psychotic disorder is developed.^5^^,^^9^ Psychotic experiences reported by young people are rarely interpreted as isolated and declared psychoses: in actual fact, they may throw light on the existence of neurotic psychopathology. Rigor in assessing the mental state and investment in a substantiated history obtained from different people and diverse sources of information, along with due respect for the potential of “adolescence” as a process of transition and maturation, appear to be of utmost importance.

Characteristically, adolescence is a period of profound change, and is thus a prime age for the onset of mental, emotional and relational disorders. During its development, the symptoms that occur leave room for doubt as to whether they refer to preclinical and prodromal signs of an existing psychotic illness or are simply physiological signs of puberty. Moments of discontinuity and rupture, emotional instability, identity crisis and intergenerational conflict are all part of normative adolescence.

In this developmental context, adolescents need to gradually acquire skills and abilities. Competence regarding self-regulation falls within this reasoning, and the relationship between the process of mood regulation and the development of psychosis appears to be of major relevance. Success in mood regulation becomes easier within a context of adequate social development that is guided by proficiency in adaptive management of disruptive situations in everyday life.

Psychopathology results from difficulties in relation to adaptive mediation of stressor circumstances, thereby inhibiting or limiting the normative development process. Symptoms may be associated with psychological conflicts within development, that are normative and transient, and/or reactive to certain situations (separation from parents, change of school, birth of a sibling, response to aggression or other traumatic events, difficulties in academic activities, difficulties in interpersonal relationships, difficulties in dealing with frustrations or in regulating emotions, etc.), and which have a dynamic and interactive component that does not appear serious and is often observed during growth.

Along this line of reasoning, two major difficulties should arise immediately in the mind of a child psychiatrist: distinguishing what is pathological from what is normative; and establishing a diagnosis when justified.

The aim of the present clinical review was to illustrate the diagnostic difficulty associated with psychotic experiences during adolescence. It was also intended to illustrate the observation that not all hallucinations occur in the context of a declared psychotic disorder. The authors describe their thoughts on psychotic experiences that can arise from a neurotic setting, and share the reasoning that was constructed in relation to the differential diagnosis.

## CASE REPORT

The patient was a 16-year-old adolescent girl in 10^th^ grade when she presented to the Emergency Department of Coimbra Pediatric Hospital (in 2013), accompanied by her father. She did not have any relevant medical history, but the death of her mother in 2010 (as a victim of breast cancer) was cited as the greatest trauma of her childhood, a circumstance that she was having difficulties in coping with.

On admission, she displayed disturbances of mood and sensory perception, concurrently with a bizarre gait abnormality. She was assessed within the neurology and pediatrics sectors, and organic pathological conditions were ruled out through analytical evaluations, electroencephalogram (EEG) and computed tomography (CT) scans. Observation within the child and adolescent psychiatry sector was then requested.

From the first observation, high levels of vegetative anxiety stood out, along with depressive mood, high levels of expressed emotion, thoughts dominated by intrusive images from the day of her mother’s funeral, changes in sensory perception (egosyntonic visual hallucinations) and behavior dominated by traits of perfectionism and obsessiveness. Her father mentioned that she was under a great deal of stress in the light of her upcoming school evaluations. No psychiatric family history was identified. A diagnosis of affective psychosis was considered, in accordance with the International Classification of Diseases, 10^th^ edition^10^ (ICD-10), and she was medicated with a low dose of an antipsychotic drug.

After about 15 asymptomatic days, the patient presented again to the Child Psychiatry Service with allopsychic disorientation, psychomotor agitation, disorganized speech broken by irrational laughter that was inconsistent with the mood, disorganized thinking, abnormalities of thought control and content, and bizarre egosyntonic visual hallucinations. A drug test on urine that was requested proved benign. After emergency administration of an antipsychotic drug, followed by short hospitalization for reassessment, a new evaluation of the patient’s mental state was made. She was found to be cognizant, cooperative and focused. Contact was reserved and she showed hypomimia. Her emotions were consistent with mild depression and blunted affect. She did not continue to show abnormalities of thought and sensory perception. Her condition was relatively similar to her previous state and she had no memory of the episode. At this stage, a diagnosis of psychotic disorder with symptoms of schizophrenia was made, in accordance with ICD-10. It was concluded from the assessment that she should be monitored long-term. Her antipsychotic medication was then adjusted, accordingly.

The improvement seen in subsequent consultations was believed to be a response to medication. However, her father confided that the first day of medication coincided with attendance at a spiritual center. Her father was understandably wondering about what was having greater impact: the medication or the spiritual center. However, because they were committed to the therapeutic process, they suspended their visits to the spiritual center.

Over the course of the follow-up her father also confided having met a lady for whom he had nurtured special feelings, prior to the onset of the clinical picture. This matter was addressed in conversations with the patient, and she reported having distrusted and disliked this relationship, because she considered that it was disrespectful to her mother’s memory.

During the treatment process, she was helped to diversify her range of strategies, and to identify and verbalize her concerns, through more adaptive management and regulation of her emotions. Structured cognitive-behavioral psychotherapy was used for an intervention regarding her traits of perfectionism and obsessiveness. Long-term observation (for about two years) of this adolescent showed favorable progression, with gradual reduction of antipsychotic medication. The antidepressant therapy was maintained, accompanied by individual and family psychotherapy. While reducing the antipsychotic medication, use of selective serotonin reuptake inhibitor (SSRI) antidepressants was gradually started and this was maintained for about a year.

Recent evaluation of her mental state revealed that the patient is now a self-aware adolescent (19-year-old) who is cooperative and focused, with very faint levels of vegetative anxiety. She was euthymic and had syntonic behavior. No abnormalities of thought or sensory perception were identified. She displayed a more developed range of coping strategies, in particular regarding management of emotions relating to her mother. There remained traces of anxious, perfectionist and hypercritical behavior, but it had clearly improved. A diagnosis of conversion disorder was suggested, in accordance with ICD-10, which gained consistency over a long-term period of monitoring. The different mental state assessments conducted can be seen in Table 1.

**Table 1:**
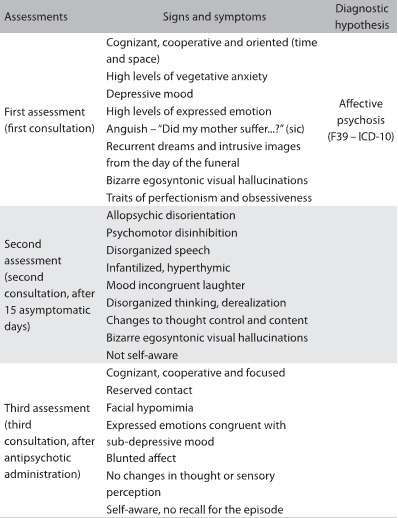
Mental state assessments

## DISCUSSION

In the present case, differential diagnoses were made between affective psychosis (F39; ICD-10), psychotic disorder with schizophrenic symptoms (F23.1; ICD-10) and conversion disorder (F44; ICD-10).

According to the ICD-10, the fundamental disturbance in affective disorders is a change in mood or behavior, usually towards depression (with or without associated anxiety) or towards elation. This change in mood is usually accompanied by a change in overall functioning and most of the accompanying symptoms are secondary or easily framed within the context of such changes. Affective psychosis falls within this context.

Around 15 days after the patient’s initial presentation, a diagnosis of psychotic disorder with symptoms of schizophrenia was suggested. According to the ICD-10, this is an acute psychotic disorder in which hallucinations, delusions and disorders are obvious, but are markedly variable, changing from day to day or even from hour to hour. Emotional turmoil with intense transient feelings of joy and ecstasy, or anxiety and irritability, are often present. Despite psychotic and emotional symptoms, there are no criteria for manic, depressive or schizophrenic episodes. Long-term observation (more than one month) is decisive.

Dissociative disorders (conversion) all involve an apparent loss that is either complete or forms part of the normal integration between memories of the past, awareness of identity and immediate sensations and control of body movements. They are presumed to be psychogenic in origin and are temporally associated with traumatic, unresolved or intolerable events, or disturbed relationships. The term “conversion” implies that there is an unpleasant effect that is engendered by the problems and conflicts that the individual cannot solve and is somehow manifested with these symptoms. These tend to be limited in time, unless they result from unresolvable problems or severe interpersonal difficulties. With regard to pathophysiology, conversion symptoms are due to unconscious repression of emotional conflicts, but there is a danger of misdiagnosis without a thorough medical workup.^11^

On admission, our patient displayed disturbance of mood and sensory perception, concurrently with a bizarre gait abnormality. She was assessed within the neurology and pediatrics sectors, and organic pathological conditions were ruled out. In the literature, conversion disorder is considered to be a relatively rare cause of walking disability. Thus, a few cases presenting alteration of gait in the context of conversion have been described.^12^^,^^13^^,^^14^^,^^15^ Conversion disorder has been found to be the third largest underlying psychological cause of psychogenic motor disorder after depression and anxiety. However, detailed descriptions of hallucinations as a conversion symptom are even rarer in the psychiatric literature. We found only a few case reports in the literature, mostly from the middle to final decades of the twentieth century.^16^^,^^17^^,^^18^^,^^19^^,^^20^^,^^21^^,^^22^^,^^23^^,^^24^ In 1982, Rack cited a case of hysterical hallucination and commented that among Asian women, especially teenagers, the commonest cause of hallucinations is hysteria and not schizophrenia.^25^ These cases shared the circumstance of involving female patients, either adolescents or young adults.

In our case, different situations of conflict were identified and managed. Therapeutic interventions were successful and the patient has recovered. Long-term observation revealed an improved state with better emotional management and a broader range of coping strategies. Remission was achieved through behavioral and psychotherapy, while antipsychotics were gradually suspended.

The patient’s condition was not considered to be part of an organically-based psychotic process. She will probably require long-term psychotherapy focusing on helping her to gain insight regarding her problems and hopefully making permanent changes for the better in both her symptoms and her personality structure.

We reviewed the literature in MEDLINE, Embase and LILACS using the English keywords “conversion disorder”, “psychotic disorder”, “schizophrenia”, “hallucinations”, “adolescent psychiatry” and “child psychiatry”, and the Portuguese keywords “transtorno conversivo”, “transtorno psicótico”, “esquizofrenia”, “alucinações”, “psiquiatria infantil” and “psiquiatria do adolescente”. The results are presented in Table 2. We only found a few reports, mostly from the middle to final decades of the 20^th^ century.

**Table 2:**
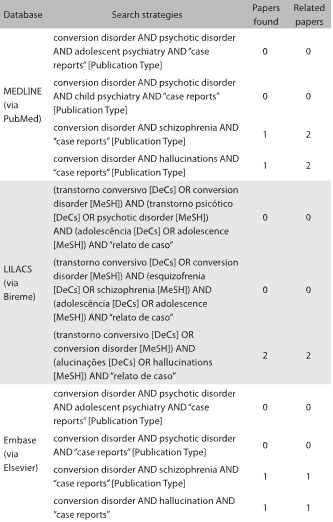
Search of the literature in medical databases for case reports on conversion disorder with hallucinations. The search was conducted on October 10, 2016

## CONCLUSIONS

Conversion disorder is considered to be a relatively rare cause of walking disability, in the literature available. However, detailed descriptions of hallucinations as a conversion symptom are even rarer, with few case reports in the literature. Our diagnostic hypothesis only gained consistency over a long-term follow-up period within the context of our child and adolescent psychiatry outpatient service, which was fundamental for appropriate diagnostic clarification. It is also important to emphasize that there is a need for rigorous evaluation of the patient’s mental state and investment in substantiated histories provided by different people and from various information sources.
